# Severity and Resolution of Diabetic Ketoacidosis in Newly Diagnosed Type 1 Diabetic Children before and During The COVID-19 Pandemic

**DOI:** 10.1900/RDS.2022.18.181

**Published:** 2022-12-31

**Authors:** Mohammad Hussain Al-Qahtani, Aqilah Taleb Al-Qassab, Fatimah Mousa Bukhamseen, Danah Zaki Al Ghanim, Sarah Hassan Alshawaf, Reem S. AlOmar, Ebtesam Abdullah Al-Suhaimi, Bassam Hassan Awary, Abdullah Abdulsalam Yousef, Waleed Hamad Albuali, Haneen Abdulsalam Yousef, Nouf A. AlShamlan

**Affiliations:** 1 Department of Pediatrics, College of Medicine, Imam Abdulrahman Bin Faisal University, Dammam, Saudi Arabia,; 2 Department of Pediatrics, Qatif Central Hospital, Qatif, Saudi Arabia,; 3 College of Medicine, Imam Abdulrahman Bin Faisal University, Dammam, Saudi Arabia,; 4 Department of Family and Community Medicine, College of Medicine, Imam Abdulrahman Bin Faisal University, Dammam, Saudi Arabia,; 5 Department of Biology, College of Science and Institute of Research and Medical Consultations (IRMC), Imam Abdulrahman Bin Faisal University, 31441 Dammam, Saudi Arabia.

**Keywords:** type 1 diabetes mellitus, diabetic ketoacidosis ·COVID-19, children

## Abstract

**OBJECTIVES:**

To epidemiologically assess the influence of COVID-19 pandemic on newly diagnosed type 1 diabetes mellitus presenting with diabetic ketoacidosis at the teaching hospital of the university, Eastern province, Saudi Arabia.

**METHODS:**

We enrolled newly diagnosed type 1 diabetes mellitus cases among pediatric patients attending the emergency department and outpatient clinics during 2019-2021. The participants’ data were collected from electronic medical records which included patients’ age at diagnosis, sex, nationality, height, weight, year of diagnosis, length of stay, presentation, random blood sugar, blood gas readings, electrolyte panel, and time of resolution of the diabetic ketoacidosis if present.

**RESULTS:**

108 patients were included with an average age of 8.87 ± 4.21 years and 53.70% were females. The demographic characteristics of all diabetic pediatric patients prior to COVID-19 and during COVID-19 were studied and the difference was statistically insignificant. Furthermore, initial pH and HCO3 tests were found to be lower in the moderate to severe diabetic ketoacidosis group (7.17 and 11.2, respectively) compared to the mild group (7.27 and 15.50, respectively) and the differences were statically significant (P < 0.001).

**CONCLUSION:**

Patients newly diagnosed with type 1 diabetes mellitus during the COVID-19 pandemic tended to have a more severe presentation of diabetic ketoacidosis in terms of PH and HCO3.

## 1 Introduction

Type 1 diabetes mellitus (T1DM) affects approximately 9.5% of children and adolescents worldwide [1]. Strict control of blood glucose level is a prerequisite to prevent the detrimental effects of hyperglycemia, which in some cases may lead to the common emergency metabolic state of diabetic ketoacidosis (DKA) [2].

External factors that play a role in the pathophysiology of DKA include dehydration, strenuous physical activities, and infection, while internal elements include the state of insulin deficiency and the counteracting hormones such as glucagon, cortisol, catecholamine’s, and growth hormone [3]. Patients with DKA present with nausea, vomiting, generalized abdominal pain, acetone breath smell, confusion, and deep respiration. Biochemically, it presents with blood glucose > 200 mg/dL, a pH of less than 7.3 or bicarbonate level less than 15mmol/L, and presence of ketonemia ≥ 3 mmol/L or moderate or large ketonuria [4]. Fluids and electrolytes such as sodium, potassium, calcium, magnesium, chloride, and phosphate are lost in urine as a result of osmotic diuresis that is induced by the state of hyperglycemia, contributing to the morbidity and mortality of this complication [3].

Internationally, a systematic study of different research databases has been conducted for publications up to March 2022. A meta-analysis was done for the comparative risk of developing new cases of T1DM and risk of DKA in Type 1 diabetic children pre- and post-the COVID-19 pandemic. In comparison with the pre-pandemic period, values of HbA1c and glucose average have risen by 6.42% and 6.43%, respectively in newly diagnosed Type 1diabetic children after the pandemic. It was found that the pandemic had markedly rose the risk of world new-onset T1DM, DKA, and severe DKA in the pediatric population [5]. In Germany, a research group from the German University Hospital studied the clinical impact of the first lockdown presentation and appearance of T1DM in children in their setting. They found that the pandemic had aggravated the complexity of T1DM onset as well treatment management, which in turn has led to recurrence and increased severity of DKA [6]. In the United States, trends in DKA among Type 1 diabetics during the pandemic at 7 major US medical centers were studied along with their associated factors and trends. DKA recurrence rose in patients with T1DM during the COVID-19 crisis with the highest ratio in non-Hispanic Black (NHB) patients. It was also found that DKA was less prevalent among those who were utilizing insulin pumps or monitored by continuous glucose monitoring (CGM) [7].

The previous studies indicate the immediate need to develop strategies and awareness for communities and physicians alike, especially those who deal with such cases including pediatricians, diabetologists and primary care physicians to avoid DKA in patients with T1DM not only during the pandemic crisis, but also in all situations particularly among populations who are majorly impacted by inequities or poor access of healthcare.

Several studies in the literature demonstrated an increase in the annual incidence of T1DM and DKA during the years of the COVID-19 pandemic as compared to the pre-pandemic era [8-11]. From a longitudinal observational retrospective study conducted at a teaching hospital in the Eastern Province of Saudi Arabia, the reported increase was from 4.17 in 2019 to 8.39 and 6.00 per 1000 in 2020 and 2021, respectively [12]. With the implications and restrictions that were implemented globally due to the COVID-19 pandemic, the prosperity of having access to healthcare services was limited for many individuals. Healthcare systems had to reallocate their resources and their available healthcare personnel to tend to those inflicted with the virus [13]. With these regulations, several patients with different illnesses presented in their disease with a worse clinical picture and higher morbidity and mortality rates than they would at a time of no global health emergency and social restrictions [14,15].

Globally, the impact of the implemented quarantine and restrictions on the newly diagnosed diabetic children with DKA and its severity were reported by many centers [9-11]. The aim of this study is to compare the presentation and severity of the newly diagnosed T1DM presenting with DKA in pre- and post-pandemic period in the Kingdom of Saudi Arabia.

## 2 Methodology

This is an observational chart review study that was carried out among children and adolescents with newly diagnosed T1DM in the pediatric emergency department and pediatric diabetes outpatient clinics at King Fahd Hospital of the University (KFHU), a teaching hospital in Al-Khobar, Eastern Province, Saudi Arabia.

The Institutional Review Board (IRB) of Imam Abdulrahman Bin Faisal University approved this study (IRB-2021-01-202). Furthermore, data confidentiality was ensured following the Declaration of Helsinki principles. The data were dealt with in an anonymous manner to protect the participants’ privacy.

To determine the impact of the COVID-19 pandemic on the clinical presentation of newly diagnosed T1DM and the severity of DKA, data were collected from medical records of patients admitted between March 2019 to September 2021 to allow for equal number of months for the two periods. This resulted in two different groups; the first one is 15 months prior to COVID-19 and the second group 15 months during COVID-19. All records were reviewed, and all patients aged less than 18 years old and were newly diagnosed with T1DM were considered for recruitment into this study.

The participants’ data were collected through electronic medical records. Patients were recognized by using the International Classification of Diseases (ICD-10) coding system which is integrated in the patients’ information system. Certain code is assigned to every medical diagnosis which corresponds to the chief complaint in each visit.

Clinical information collected included patients’ age at diagnosis, sex, nationality, height, weight, year of diagnosis, length of stay, random blood sugar, initial blood gas readings and initial electrolyte panel (NA, K, CL, PO4). For patients presenting with DKA additional parameters were included such as corrected arterial blood gas readings, corrected electrolyte panel, and time of resolution of DKA.

**Figure 1.**
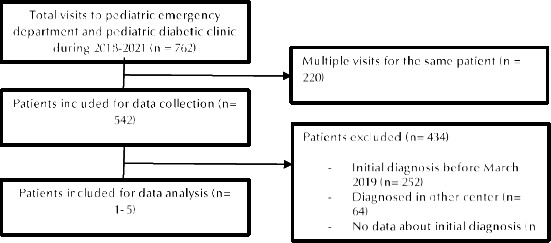
Flowchart of included participants.

DKA was defined based on blood gas readings of HCO3 <16 and pH <7.35. The time to achieve DKA resolution is the time between the patient’s admission until the time when the pH is >7.35 and or HCO3 >16. Severity of DKA was classified according to the International Society for Pediatric and Adolescent Diabetes (ISPAD) into; mild, moderate, and severe (Table 1). A total of 542 patients were initially enrolled. We excluded all patients diagnosed in other centers as well as those who had missing data regarding their initial diagnosis or incorrect clinical code (not T1DM) (Figure 1).

**Table 1.** Severity categorization of diabetic ketoacidosis

	Mild	Moderate	Severe
pH	pH 7.34 -7.25	pH 7.24 -7.15	pH < 7.15
Hco3	HCO3 15-10	HCO3 5 - 9	HCO3 < 5

### 2 1 Statistical analysis

Continuous data were described as means ± standard deviation or me dian and interquartile range (IQR) depending upon their normality. Categorical data were described as counts and percentages. Comparative analyses were done through a series of t-tests when the continuous variable was normal, or Man-Whitney U tests if the continuous variable was non-normal. Cross tabulation through Chi-squared tests and Fisher’s exact were performed to obtain p-values as well. Due to the small number of end points, regression analyses were not performed. The level of significance for p-values was set to 0.05. All analyses were performed in Stata Software version 15.1.

## 3 Results

### 3.1 Overall patient characteristics

A total of 108 pediatric patients had presented to the hospital between March 2019 and September 2021. Overall, patients had an average age of 8.87± 4.21 years. Females were more than males accounting for 53.70%. More importantly, the ones presenting with DKA made up 44.44% of the entire data spanning both periods (Table 2).

**Table 2.** Demographic characteristics of all diabetic pediatric patients prior to COVID-19 and during COVID-19

Characteristics	Total	Pre-COVID-19	During COVID-19	P-value
	108 (100.00)	51 (47.22)	57 (52.78)	
**Age (X, σ_x_)**	8.87 (4.21)	8.58 (4.32)	9.14 (4.13)	0.75
**Gender**				
Males	50 (46.30)	23 (46.00)	27 (54.00)	0.67
Females	58 (53.70)	29 (50.00)	29 (50.00)	
**Nationality**				
Saudi	101 (93.52)	49 (48.51)	52 (51.49)	0.54
Non-Saudi	7 (06.48)	3 (42.86)	4 (57.14)	
**Patient had DKA**				0.15
No	60 (55.56)	32 (53.33)	28 (46.67)	
Yes	48 (44.44)	19 (39.58)	29 (60.42)	
**Length of Stay (days)**	3 (1, 3)	2 (1, 3)	3 (1, 4)	0.35

### 3.2 Pre-COVID and during COVID-19 comparisons for all diabetic pediatric patients

The data shows that the number of patients presenting with diabetes was slightly more during the COVID-19 period (52.78%). During COVID-19, patients tended to be older when compared to the pre-COVID period (mean age = 9.14 ± 4.13 and 8.58 ± 4.32 respectively), although this difference was not statistically significant. The length of stay was higher for patients during COVID-19 too (Table 2).

### 3.3 Pre-COVID and during COVID-19 comparisons for pediatric patients presenting with DKA

Further examination of pediatric patients presenting with DKA has found that 55.36% of them were females and that the overall majority had only mild DKA (58.93%). However, upon comparison between pre-COVID-19 and during COVID-19, for both levels of severity most patients presented during COVID-19 indicating a non-statistically significant difference. Additionally, the IQR of the length of stay during the pandemic was relatively higher than the pre-pandemic period (IQR = 3 – 4 and IQR = 2 -3 respectively). However, these differences were not statistically significant (Table 3).

**Table 3.** Comparative analysis of pediatric patients presenting with DKA prior to COVID-19 and during COVID-19

Characteristics	Total 56 (100.00)	Pre-COVID 23 (41.07)	During COVID-19 33 (58.93)	P-value
**Age (X, σ_x_**	8.87 (4.21)	9.30 (4.57)	9.36 (4.08)	0.52
**Gender**				0.27
Males	25 (44.64)	8 (32.00)	17 (68.00)	
Females	31 (55.36)	15 (48.39)	16 (51.61)	
**Nationality**				0.65
Saudi	54 (96.43)	22 (40.74)	32 (59.26)	
Non-Saudi	2 (03.57)	1 (50.00)	1 (50.00)	
**Level of DKA**				0.17
Mild	33 (58.93)	16 (48.48)	17 (51.52)	
Moderate to severe	21 (41.07)	7 (30.43)	16 (69.57)	
**length of Stay (days)**	3 (2, 4)	3 (2, 3)	3 (3, 4)	0.07

**Figure 2.**
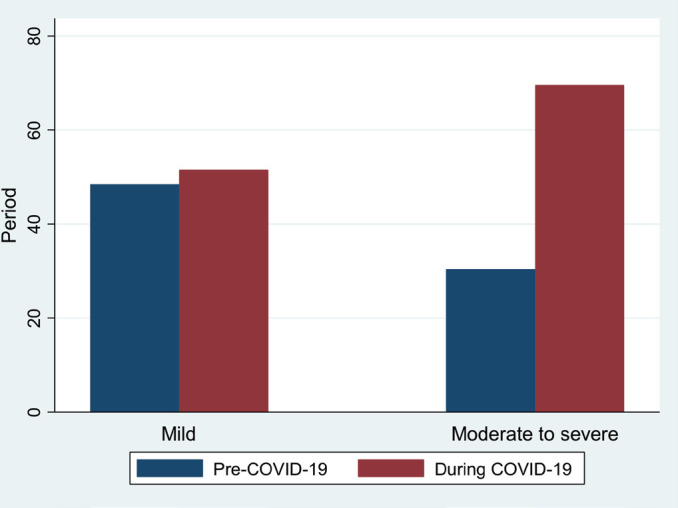
Level of severity of DKA prior to COVID-19 and during COVID-19.

Figure 2 presents the levels of severity of DKA during both periods which shows the stark difference in the moderate to severe DKA between the examined time periods.

Table 4 presents the baseline clinical characteristics of patients with DKA according to the level of severity. Statistically significant differences were observed for PH and HCO3 tests which were found to be lower in the moderate to severe group compared to the mild group (P < 0.001). Further significant differences were observed for CO2 and CI tests. Regarding the time to DKA resolution, the median time was found to be slightly higher for the mild group (Median = 16.81, IQR = 9.52 – 23.28) when compared to the moderate to severe group (Median = 16.37, IQR = 10.25 – 23.50). This difference however was not statistically significant.

**Table 4.** Baseline characteristics of pediatric patients presenting with DKA according to its level of severity

Baseline clinical readings (Median, IQR)	Total	Mild	Moderate to severe	P-value
**Initial PH**	7.30 (7.19, 7.37)	7.27 (7.15, 7.30)	7.17 (6.99, 7.21)	<0.001
**Initial HCO3**	19.40 (11.70, 23.10)	15.50 (11.50, 18.70)	11.2 (7.8, 13.3)	<0.001
**Initial CO2**	37.10 (27.10, 41.90)	30.40 (26.60, 38.40)	26.6 (19.9, 33.8)	0.01
**Initial blood sugar**	409 (338, 497)	378 (324.5, 505.5)	432 (381, 515.5)	0.10
**Initial K**	4.30 (4.00, 4.70)	4.30 (3.90, 4.70)	4.20 (3.70, 4.70)	0.64
**Initial Na**	134 (131, 137)	134 (131, 136)	136 (132, 138)	0.14
**CI**	99 (95.50, 102.50)	98 (94, 102)	103 (100, 107)	0.006
**Initial P**	4.50(3.90, 5.00)	4.20 (3.55, 4.60)	3.90 (3.40, 4.70)	0.49
**DKA resolution (hrs)**	16.81 (10, 24)	16.81 (9.52, 23.28)	16.37 (10.25, 23.50)	0.96
**Length of Stay**	3 (2, 4)	3 (2, 4)	3 (3, 6)	0.16

**Figure 3.**
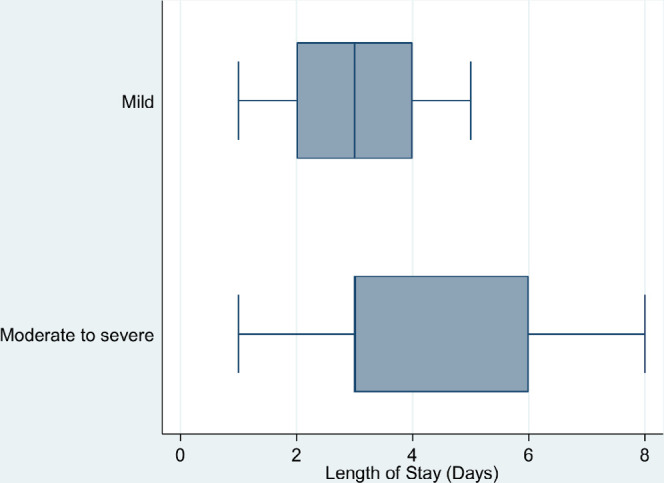
Length of stay in days according to the level of severity of DKA in pediatric patients.

Figure 3 shows the distribution of the length of stay in days according to the level of severity. Although the median was 3 days for both levels, the variance in days was much larger for the moderate to severe group indicating a higher window of longer length of stay for some of these patients.

## 4 Discussion

DKA is a life-threatening complication and a common first-presentation of T1DM. It is a timesensitive diagnosis which necessitates early recognition to ameliorate the severity of this condition. It has been linked to poor prognosis in terms of HbA1C readings, and subsequently microvascular complications, in newly diagnosed patients [16]. Nonetheless, COVID-19 pandemic has greatly impacted healthcare systems and molded lifestyles to prevent the spread of this virus. Access to healthcare has been altered by the relocation of resources to serve COVID-19 patients and the aversion of patients from attending to healthcare facilities to minimize their exposure [17]. In our chart review study, we examined newly diagnosed T1DM patients presenting through the year 2019-2021, to assess the influence of these factors on newly diagnosed T1DM presenting with DKA.

In this paper, our findings showed an increase in the number of newly diagnosed patients presenting with DKA; however, this difference was not statistically significant. Like our results, Kostopoulou et al results indicated a statistically insignificant increase in their numbers from 35.3% to 66.7% during pre-COVID-19 and COVID-19 pandemic, respectively [18]. In contrast, several articles published have established a significant increase in the incidence of DKA among newly diagnosed patients. A study conducted in Poland demonstrated a significant rise in the percentage of DKA among newly diagnosed patients [19]. Kamrath et al findings also exhibited a significant increment of around 20% in DKA presentations among newly diagnosed patients [20]. In addition, a Saudi multicentric retrospective study involving middle, western and southern regions of the country, showed an increase in DKA incidence rate among newly diagnosed patients by around 13% [8]. Meanwhile, a study conducted in a tertiary hospital in Seattle, in the United States, showed a stable number of newly diagnosed patients with DKA before and after the pandemic [17].

To assess the severity of DKA, parameters such as blood gasses, electrolytes, and glucose were all recorded and analyzed in our study. Our data showed a trend of lower pH and HCO3 among moderate to severe DKA patients when compared to mild DKA patients. These results were comparable to the international literature published. Studies conducted in Greece, US, and Germany all confirmed that DKA presentation during the COVID-19 period was significantly more severe, with higher pediatric intensive care unit (PICU) admission rates [9,18,20]. Similarly, Jafari has established that during the stay-at-home orders in the United States pH readings tended to be lower than usual [17]. Moreover, females and older children were found to be at a higher risk of DKA [8,20].

Throughout our study period, the average length of stay (LOS) in hospital was 3 days in both mild and moderate to severe DKA. However, moderate to severe DKA had a higher window of longer length of stay for some of these patients in comparison with mild DKA (3-6 vs 2-4 days), respectively. Our results were consistent with national and international studies which were conducted to assess the impact of COVID-19 pandemic on the severity of DKA among the pediatric population. A multicentric retrospective cohort study in Saudi Arabia conducted by Alaqeel et al reported a higher length of stay in intensive care units in 2020 compared to 2019 (0.5 vs 0.6 days), respectively [8]. Similarly, a retrospective cohort study that involved pre-pandemic and pandemic patients who were admitted with DKA to a 30-bed PICU in the United States reported that pandemic patients had a longer PICU length of stay than pre-pandemic period patients (1 Vs 0.75 days), respectively [9]. The study determined that pediatric patients whose parents felt that COVID-19 pandemic limited their ability to attend healthcare centers had a more severe DKA and therefore stayed longer in PICU [9].

### 4.1 Limitation

Our study has a few limitations. First, because of the retrospective nature of our study, not all data can be collected due to incomplete medical reports. During the COVID-19 pandemic, most patients tended to avoid presenting to the healthcare center in person to diminish the exposure to COVID-19. This contributed to a lower rate of laboratory work-up for this population. Eventually, the number of patients in this study was limited. Furthermore, this was a single center-based study so the result cannot be generalized to other settings. Larger public health population studies are needed and recommended to further determine the factors that might affect the incidence of DKA and its severity during the COVID-19 era.

### 4.2 Conclusion

The current study was able to give an epidemiological insight into DKA severity, which was assessed clinically and by presenting pH and HCO3 readings and found that it had worsened during the COVID-19 pandemic period. The pandemic had potentially altered the access to healthcare centers which in turn delayed resulted in delaying the diagnosis of diabetes which markedly increased DKA frequency and severity. Therefore, physicians should consider this issue in all pediatric patients with new onset symptoms suggestive of T1DM and monitor their random blood sugar and HbA1C when they present with nonspecific symptoms for proper and early management. This data emphasizes the importance of repeatedly conducting diabetes awareness campaigns for physicians, caregivers, and the public to improve the healthcare outcomes in such a significantly common and potentially serious disease.

## Abbreviations

T1DM: Type 1 Diabetes mellitus

COVID-19: Corona Virus Disease 2019

DKA: Diabetic Ketoacidosis

ICD: International Classification of Disease

PICU: Pediatric Intensive Care Unit

HbA1c: Glycosylated Hemoglobin A1c
